# Marine biofilms on different fouling control coating types reveal differences in microbial community composition and abundance

**DOI:** 10.1002/mbo3.1231

**Published:** 2021-08-24

**Authors:** Maria Papadatou, Samuel C. Robson, Sergey Dobretsov, Joy E. M. Watts, Jennifer Longyear, Maria Salta

**Affiliations:** ^1^ School of Biological Sciences University of Portsmouth Portsmouth UK; ^2^ School of Pharmacy and Biomedical Sciences University of Portsmouth Portsmouth UK; ^3^ Centre for Enzyme Innovation University of Portsmouth Portsmouth UK; ^4^ Department of Marine Science and Fisheries College of Agricultural and Marine Sciences Sultan Qaboos University Muscat Oman; ^5^ Centre of Excellence in Marine Biotechnology Sultan Qaboos University Muscat Oman; ^6^ AkzoNobel/ International Paint Ltd Felling, Gateshead UK

**Keywords:** antibiofilm strategies, biofilms, biofouling, fouling control coatings, microbial community

## Abstract

Marine biofouling imposes serious environmental and economic impacts on marine applications, especially in the shipping industry. To combat biofouling, protective coatings are applied on vessel hulls which are divided into two major groups: biocidal and non‐toxic fouling release. The current study aimed to explore the effect of coating type on microbial biofilm community profiles to better understand the differences between the communities developed on fouling control biocidal antifouling and biocidal‐free coatings. Biocidal (Intersmooth® 7460HS SPC), fouling release (Intersleek® 900), and inert surfaces were deployed in the marine environment for 4 months, and the biofilms that developed on these surfaces were investigated using Illumina NGS sequencing, targeting the prokaryotic 16S rRNA gene. The results confirmed differences in the community profiles between coating types. The biocidal coating supported communities dominated by Alphaproteobacteria (*Loktanella*, *Sphingorhabdus*, *Erythrobacter*) and Bacteroidetes (*Gilvibacter*), while other taxa, such as *Portibacter* and Sva0996 marine group, proliferated on the fouling‐release surface. Knowledge of these marine biofilm components on fouling control coatings will serve as a guide for future investigations of marine microfouling as well as informing the coatings industry of potential microbial targets for robust coating formulations.

## Graphical abstract

Marine biofilms constitute the pioneering components of biofouling, a phenomenon with significant environmental and economic impacts in maritime applications. Antifouling control strategies are applied on vessel hulls based on the application of protective paints. Here, we explored the in situ prokaryotic biofilm community composition and abundance on different types of commercial fouling control coatings (fouling release, biocidal) employing next‐generation sequencing targeting the 16S rRNA gene. Our results indicated distinct microbial community profiles between coating types, with specific genera contributing to differences between surfaces.

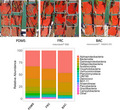

## 1 INTRODUCTION

Biofouling is the undesirable accumulation of microorganisms, animals, and plants on immersed structures in aquatic habitats (Railkin, 2004), an omnipresent and highly dynamic phenomenon (Harder, 2008; Holmström et al., 2006). Aquatic biofilms are the pioneering components of the biofouling process (Salta et al., 2013; Wahl, 1989) and constitute assemblages of microbial cells irreversibly attached to living or non‐living surfaces, embedded in a self‐produced matrix of hydrated extracellular polymeric substances (EPS) (Costerton, 1999; Zobell & Allen, 1935). Biofouling constitutes a significant issue in maritime industries, and problems related to microfouling include an increase in drag force, modification of surface properties, and production of chemical cues (Dobretsov et al., 2013). To combat biofouling in the shipping industry, fouling control coatings are applied to ships’ hulls where the biofilms first attach (Finnie & Williams, 2010). These commercial fouling control coatings are either biocidal antifouling or non‐biocidal fouling‐release coatings.

Biocidal antifouling coatings function through the release of certain toxic chemicals (biocides) to deter the settlement and growth of organisms. Biocidal coatings remain the most popular choice and still dominate the market reportedly accounting for more than 90% of coatings sales (Lejars et al., 2012; Winfield et al., 2018), although concerns over the potential environmental impact of biocides have led to increased attention being paid to the development of biocide‐free approaches to fouling control (Lejars et al., 2012). Non‐biocidal fouling‐release coatings function based on low surface energy, smooth and non‐porous, free of reactive functional groups (Finnie & Williams, 2010) which reduces an organism's ability to generate a strong interfacial bond with the surface (Chambers et al., 2006; Lejars et al., 2012). Thus, such coatings minimize the adhesion strength of organisms and facilitate their removal either by hydrodynamic forces (water flow) as the vessel moves or through their organisms’ weight (Swain, 1999). Both coating types have been shown to bear similar low levels of fouling after extended immersion if exposed to flow levels comparable to slow ship speeds, with the fouling‐release coating losing most accumulated biomass in flow, consistent with its low adhesion mechanism of action (Davidson et al., 2020). Fouling‐release coatings have a smaller market share when compared to biocidal since they generally require flow to be effective against biofouling (Briand et al., 2012; Molino et al., 2009). Although the coating industry has an increasing interest in the development of biocide‐free (micro)fouling control solutions that rely on surface physico‐chemical properties, the development of a successful marine coating that is simultaneously effective against biofouling while being substantially environmentally benign is very challenging.

Biofilm research is important to the marine coating industry as it directly provides insights into the response of biofilm communities on coating surfaces and consequently may inform the development of new paint technologies. Several studies investigated the effect of fouling control coatings on *in situ* biofilm community composition either by employing light and epifluorescent microscopy (Cassé & Swain, 2006), or molecular fingerprinting and microscopic observations (Briand et al., 2012), or flow cytometry coupled with denaturing gradient gel electrophoresis and light microscopy (Camps et al., 2014); all of which have reported that the observed biofilm community compositions were influenced by coating type.

To date, only a handful of studies have reported the application of next‐generation sequencing (NGS) techniques to investigate the response of marine biofilm community profiles developed on marine fouling control coatings (von Ammon et al., 2018; Briand et al., 2017; Ding et al., 2019; Dobretsov et al., 2019; Flach et al., 2017; Hunsucker et al., 2018; Leary et al., 2014; Muthukrishnan et al., 2014; Winfield et al., 2018). NGS technology based on sequencing ribosomal RNA genes is appropriate for a range of applications including highly diverse community analysis while offering a large volume of data that allow for statistical testing (Fukuda et al., 2016). All studies employing high‐throughput NGS have reported the dominance of Alphaproteobacteria on fouling control coatings, while Gammaproteobacteria have also been identified as key players in fouling control systems (von Ammon et al., 2018; Briand et al., 2017; Ding et al., 2019; Dobretsov et al., 2019; Flach et al., 2017; Hunsucker et al., 2018; Leary et al., 2014; Muthukrishnan et al., 2014). Biofilms on fouling control coatings have also been dominated by Flavobacteria (von Ammon et al., 2018; Hunsucker et al., 2018; Leary et al., 2014; Muthukrishnan et al., 2014) or Cyanobacteria (Ding et al., 2019; Hunsucker et al., 2018; Leary et al., 2014; Muthukrishnan et al., 2014). To a smaller extent, the prevalence of Planctomycetes (von Ammon et al., 2018; Ding et al., 2019; Leary et al., 2014) and Verrucomicrobia (Leary et al., 2014; Winfield et al., 2018) have also been reported. Taking into account the rapid advances in sequencing technologies, it is essential to generate up‐to‐date NGS studies investigating biofilms on fouling control surfaces. Despite the current knowledge, certain aspects of biofilm research on fouling control coatings remain elusive. Differences in biofilm profiles between biocidal and fouling control coatings can help to highlight potential targets of importance for effective antibiofilm control, as well as identifying potential biocidal‐tolerant biofilm components at low taxonomic levels.

The aims of the present study are (1) to explore and characterize marine biofilm communities isolated from commercial fouling control coatings using 16S rRNA gene amplicon sequencing and (2) to compare the biofilm profiles developed between fouling release and biocidal coating types. To reflect the biofilm formation based on state‐of‐the‐art analyses and study design, a combination of biocidal antifouling coating, fouling‐release coating, and reference surfaces were used, testing *in situ* four biological replicates of biofilms using Illumina MiSeq sequencing targeting the V4‐V5 region of the 16S rRNA gene to examine bacterial composition. The purpose of this work is to elucidate biofilm components at the genus level that are selectively attached on biocidal and/or fouling‐release surfaces. The study findings will contribute knowledge into the growing body of NGS studies of biofilms on fouling control paints and subsequently inform the future design of fouling control surfaces.

## 2 MATERIALS AND METHODS

### 2.1 Commercial fouling control coatings

Three treatments were exposed during the immersion study including (1) a commercial biocidal antifouling coating which will be termed as “BAC” (Intersmooth® 7460HS SPC, self‐polishing copolymer coating that contains cuprous oxide and copper pyrithione biocides), (2) a commercial non‐biocidal fouling‐release coating which will be termed as “FRC” (Intersleek® 900, fluoropolymer), and (3) a non‐biocidal inert surface termed as “PDMS” (silicone paint film incorporating a generic unmodified polydimethylsiloxane matrix). A red pigmentation was incorporated in all coated panels to minimize the potential influence of surface color on community variation. Details of all surfaces are presented in Appendix Table A1.

Experimental panels were prepared by brush application at the International Paint Laboratories in Gateshead UK following the correct scheme for each coating type (BAC: anticorrosive primer plus finish coat; FRC, PDMS: anticorrosive primer, silicone tie coat, finish coat). The panels were double‐side‐coated with dimensions 8.5 × 8.5 cm^2^.

### 2.2 Panel deployment and study site

**FIGURE A1:**
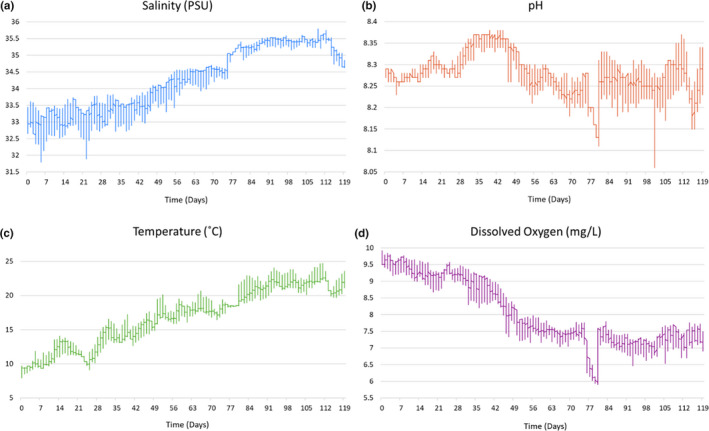
Environmental parameters throughout the 4‐month coatings deployment, including (a) salinity (PSU), (b) pH, (c) temperature (˚C), (d) dissolved oxygen (mg/L). Records were concluded using sensor YSI 6820V2 sonde located in the Institute of Marine Science, University of Portsmouth

Experimental panels were attached to a metal frame using cable ties and deployed to the anchored University of Portsmouth (UoP) raft (50°48'23.4"N 1°01'20.1"W) in Langstone Harbour, UK. Frames were immersed vertically to the seawater surface at 0.5 – 1 m depth for 119 days from April 6 until August 3, 2018 (Appendix Figure A1).

The sampling location is characterized as a semi‐diurnal system, where two high and two low tides take place every 24 hours. It has a temperate climate moderated by prevailing southwest winds and significant rainfall. Langstone Harbour entrance is characterized by maximum spring tide flood velocities of 0.9 ms^−1^ (1.7 knots), ebb velocities 1.8 ms^−1^ (3.5 knots), and mean flood tidal stream velocity of 0.7m^s−1^ (1.4 knots) (www.scopac.org.uk, www.eoceanic.com).

### 2.3 Biofilm sample collection and storage

**FIGURE A2:**
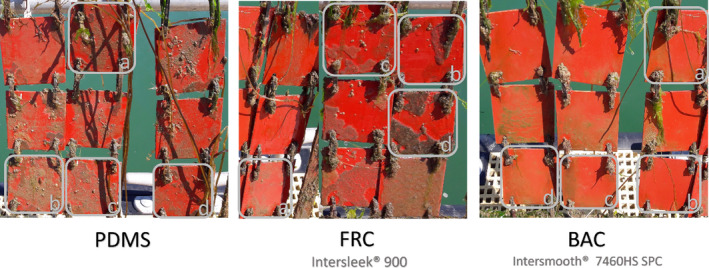
Visual observation of endpoint biofilm samples before collection of fouling control coatings; PDMS, FRC, and BAC for DNA extraction. The gray squares indicate the individual replicate sample of each surface type

The biofilms samples were collected (*n* = 4 per coating) from panels using sterile swabs (Appendix Figure A2). Macrofoulers were removed from heavily fouled panels using sterile forceps. The swab was passed 10 times over the panel with circular movements for biofilm collection. During sampling, the frames were manually removed from the seawater and exposed to air during collection, for approximately 5–15 min. Between sampling, all panels were hydrated with surrounding seawater. After biofilm collection, each swab was placed into a sterile Eppendorf tube and the breakpoint was cut out using sterile scissors. Samples were then immediately snap‐frozen in liquid nitrogen (in the field), transferred to the laboratory, and stored at −80 ˚C within 4 hours. DNA extraction took place within 2 months of sampling.

### 2.4 DNA extraction and quantification

Genomic DNA (gDNA) was extracted using the DNeasy PowerBiofilm Kit (QIAGEN). The samples were transferred from −80 ˚C to room temperature. In a laminar flow hood, each biofilm swab sample was placed into a PowerBiofilm Bead Tube using sterile forceps. Qiagen's protocol (DNeasy® November 2016) was followed according to the manufacturer's instructions, except the first step was omitted, since the saturated biofilm material was attached to the swab; therefore, no weighing and centrifugation was applicable. To bead‐beat, the sample, a PowerLyzer 24 Homogenizer (MP Biomedical, FastPrep‐24™ 5G) was used. At the final step, extracted DNA was eluted following the manufacturer's instructions and stored in −80 ˚C. The quantity and partial quality of nucleic acid samples were assessed based on absorbance spectrums using a spectrophotometer (Thermo Scientific, NanoDrop 1000).

### 2.5 Next‐generation Sequencing

Twelve lyophilized gDNA samples (50 μL) were supplied to the Molecular Research DNA Lab (www.mrdnalab.com, Shallowater, TX USA). PCR conditions consisted of initial denaturation at 94℃ for 3 minutes, followed by 30 cycles of 94℃ for 30 seconds, 53℃ for 40 seconds, 72℃ for 1 minute, and the final elongation step at 72℃ for 5 minutes. Following amplification, PCR amplicon products were visualized in a 2% agarose gel. Multiple samples were pooled together in equimolar concentrations based on their molecular weight and DNA concentrations and purified using calibrated Ampure XP beads.

High‐throughput amplicon sequencing covering the V4‐V5 region of the 16S rRNA gene was performed on an Illumina Miseq 2 × 300 paired‐end platform (Illumina, San Diego, CA USA) using the universal primers 515F (GTGYCAGCMGCCGCGGTAA) and 926R (CCGYCAATTYMTTTRAGTTT) (Parada et al., 2016) following the manufacturer's guidelines.

### 2.6 Bioinformatic analyses

Raw sequence data were trimmed using Trim Galore (Babraham Bioinformatics, Cambridge UK) with parameters *‘*‐‐*illumina* ‐*q 20* ‐‐*stringency 5* ‐*e 0*.*1* ‐‐*length 20* ‐‐*trim*‐*n’*. Filtered reads were processed in QIIME2 (Bolyen et al., 2019) using the standard 16S rRNA gene amplicon analysis pipeline. Briefly, paired reads were joined, denoised using ‘qiime dada2 denoise‐paired‘, and sequences were clustered into operational taxonomic units (OTUs) that were annotated against the SILVA SSU 132 database (Pruesse et al., 2012; Quast et al., 2013; Yilmaz et al., 2014) by clustering at 99% sequence similarity cutoff (1% divergence).

The generated OTU table was then analyzed using the *R* programming language (version 4.0.2) (R Core Team, 2020). The phylogenetic analysis was implemented using the *phyloseq* package (McMurdie & Holmes, 2013) available as part of the Bioconductor project (Gentleman et al., 2004), which supports OTU‐clustering formats and provides ecology and phylogenetic tools. Sequences detected with high similarity to chloroplast and mitochondria from the eukaryotic component of the community were removed from the analysis. Plots were generated using the *ggplot2* library (Wickham, 2016).

### 2.7 Statistical analyses

Statistical tests were performed in *R*. The significance of coating type on the resulting diversity indices (Chao1, Shannon) was assessed by ANOVA (sum of squares type II), followed by the estimated marginal means (EMMs) to identify significant differences between pairwise comparisons.

Biofilm community structure (relative abundance) of phyla, classes, families, and genera was evaluated for changes between coating types using analysis of similarities (ANOSIM) (Clarke, 1993) in the *vegan R* package (Oksanen et al., 2019) with Bray–Curtis of 9999 permutations. To determine finer resolution taxa (genus level) that significantly contribute to differences between coating samples diversity (shown in ANOSIM), similarity percentages analysis (SIMPER) (Clarke, 1993) in vegan was performed using *kruskal*.*pretty* function (Steinberger, 2016) for Kruskal–Wallis tests of multiple comparisons. OTUs were deemed significant and presented for genera that contribute at least >1.5% of the variance between at least one pairwise comparison with Kruskal *p*‐value <0.05.

## 3 RESULTS

### 3.1 Quality of biofilm OTUs revealed with Illumina MiSeq sequencing

The 16S rRNA gene dataset recovered from amplicon sequencing of the V4‐V5 region using Illumina MiSeq resulted in 2,409,154 raw total sequences of 251 base pair length. The final filtered dataset consisted of 1,451,982 read pairs, with coverage ranging from 73,764 for sample PDMS_b to 215,665 for sample BAC_a (Table 1). The average number of filtered read pairs per sample was 91,918 for PDMS, 100,498 for FRC, and 170,580 for BAC.

**TABLE 1:** Characteristics of replicated biofilm samples including the sample type where biofilms were collected from, DNA concentration and quality ratios, the number of reads retrieved and assigned OTUs

Sample Type	Replicate	Concentration (ng/mL)	260/280 ratio	260/230 ratio	Filtered read pairs	OTU abundance	OTUs
PDMS	a	115.2	1.93	2.14	76,850	24,018	255
PDMS	b	91.2	1.83	1.30^a^	73,764	19,833	213
PDMS	c	63.7	1.86	0.70 ^a^	97,941	32,263	384
PDMS	d	100.0	1.85	1.66	119,116	39,773	403
average					91,918	28,972	314
FRC	a	26.0	1.92	0.83 ^a^	83,046	25,663	270
FRC	b	29.7	1.67 ^a^	0.19 ^a^	108,840	24,330	199
FRC	c	62.5	1.87	1.42 ^a^	105,287	29,855	283
FRC	d	67.5	1.82	1.24 ^a^	104,818	31,555	308
average					100,498	27,851	265
BAC	a	65.4	1.95	1.22 ^a^	215,665	19,644	101
BAC	b	41.7	1.75 ^a^	1.14 ^a^	144,125	15,959	96
BAC	c	204.2	1.86	2.04	171.618	16,445	90
BAC	d	207.6	1.87	1.89	150,912	15,297	98
average					170,580	16,836	96
Total					1,451,982		

^a^ These samples values did not meet the suggested criteria for optimal sample quality: (i) DNA yield level above 20 ng/μl, (ii) 260/280 ratio between 1.8–2.1, (iii) 260/230 ratio above 1.5, as suggested by Peimbert and Alcaraz (2016).

Following processing and clustering at the 99% sequence similarity, the 12 biofilm samples produced a total of 2,113 distinct OTUs. The average number of OTUs per sample was 314 for PDMS, 265 for FRC, 96 for BAC. The average number of OTU abundance per sample was 28,972 for PDMS, 27,851 for FRC, and 16,836 for BAC (Table 1).

### 3.2 Biofilm diversity analysis

#### 3.2.1 Alpha diversity

**FIGURE A3:**
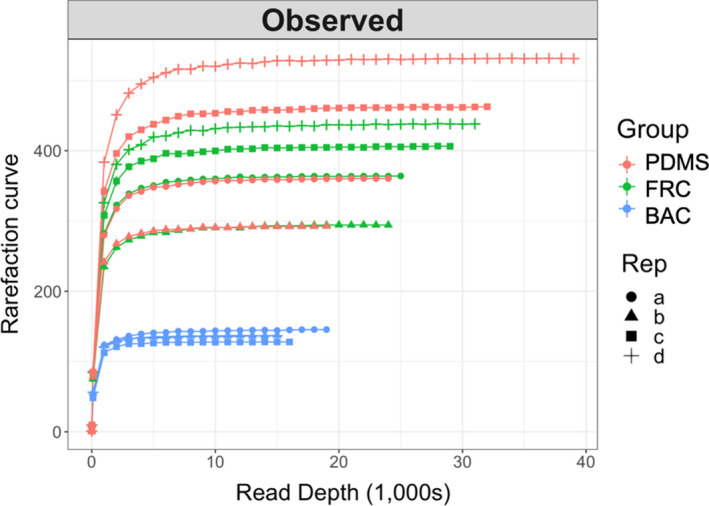
Rarefaction curves of observed alpha diversity across a range of sub‐sequencing depths for all replicates of PDMS, FRC, and BAC samples from 16S rRNA amplicon sequencing. In the present dataset, rarefaction curves for all replicates of PDMS, FRC, and BAC samples reached saturation level (Figure S3), which indicated that the current sequencing depth (Table 1) was sufficient to provide a representative diversity for these biofilm samples. The results show that BAC curves reached a horizontal asymptote at a smaller depth (~15,000 OTUs); hence, it can be inferred that a smaller number of reads could potentially reflect a good representation of the total biofilm community diversity for BAC samples, compared with PDMS or FRC that require a higher number of reads

The alpha diversity indices were calculated after rarefication to 15,000 OTU depth (per sample) (Table 2, Appendix Figure A3). At the 15,000 OTU depth, the Chao1 index varied for the individual samples between 128 (sample BAC_c) and 531 (sample PDMS_d), with the lowest values found consistently in the BAC sample (Table 2). The average Chao1 per sample type was 412 for PDMS, 376 for FRC, and 137 for BAC. Since at the 15,000 OTU depth, the Shannon index ranged between 4.13 and 6.01, with the lowest average values observed in BAC samples and the highest in PDMS samples (Table 2). The results demonstrate that BAC samples exhibited a lower diversity abundance and evenness compared to the FRC or PDMS samples and possibly encountered fewer rare species.

**TABLE 2:** Alpha diversity indices (a) for all replicate samples per treatment rarefied to 15,000 OTU depth and (b) for averaged samples per treatment rarefied to 15,000, 10,000, 1,000 OTU depths

sample type	Replicate	OTU depth	Observed diversity	Chao1	Shannon
(a)					
PDMS	a	15,000	359.80	360.82	5.55
PDMS	b	15,000	292.00	292.76	5.35
PDMS	c	15,000	458.10	462.60	5.84
PDMS	d	15,000	527.60	531.35	6.01
FRC	a	15,000	362.00	363.45	5.59
FRC	b	15,000	292.90	294.47	5.26
FRC	c	15,000	404.30	407.30	5.67
FRC	d	15,000	434.90	437.93	5.77
BAC	a	15,000	144.90	146.11	4.33
BAC	b	15,000	137.20	137.68	4.21
BAC	c	15,000	128.00	128.00	4.44
BAC	d	15,000	136.40	136.98	4.13

Sample Type	OTU depth	Observed diversity	Chao1	Shannon
(b)				
PDMS	15,000	409.38	411.88	5.69
PDMS	10,000	405.53	411.51	5.68
PDMS	1,000	309.83	366.26	5.48
FRC	15,000	373.53	375.79	5.57
FRC	10,000	370.65	374.94	5.57
FRC	1,000	288.20	336.86	5.39
BAC	15,000	136.63	137.20	4.28
BAC	10,000	136.13	137.01	4.27
BAC	1,000	119.43	127.28	4.20

The alpha diversity indices calculated at lower sub‐sampling depths, that is, 10,000 and 1,000 displayed consistent patterns with the maximum OTU count identified for all replicates (15,000). Overall, BAC replicate samples showed the lowest observed diversity, Chao1 and Shannon indices (Table 2) confirmed by the number of OTUs (Table 1), while FRC and PDMS samples were characterized by higher and close scores. Notably, BAC samples exhibited the highest number of raw reads (highest coverage) compared to the other two surfaces (Table 1). These contrasting results confirm that the low diversity of BAC samples in the present dataset is not a result of potential low sequence coverage, but rather the presence of few very abundant biofilm taxa.

A significant difference between Chao1 estimates in different treatments at the 15,000 OTU depth was shown (*p* = 0.0003***, *F* = 23.62) and the pairwise comparisons revealed significant difference between BAC – PDMS (*p* = 0.0004) and BAC – FRC (*p* = 0.0007) but not for PDMS – FRC (*p* = 0.915). The same tests for Shannon diversity index showed significant difference between sample types (*p* = 0.0002***, *F* = 24.5), and the pairwise comparisons revealed significant difference between BAC – PDMS (*p* = 0.0005) and BAC – FRC (*p* = 0.0005) but not for PDMS – FRC (*p* = 1).

**FIGURE 1:**
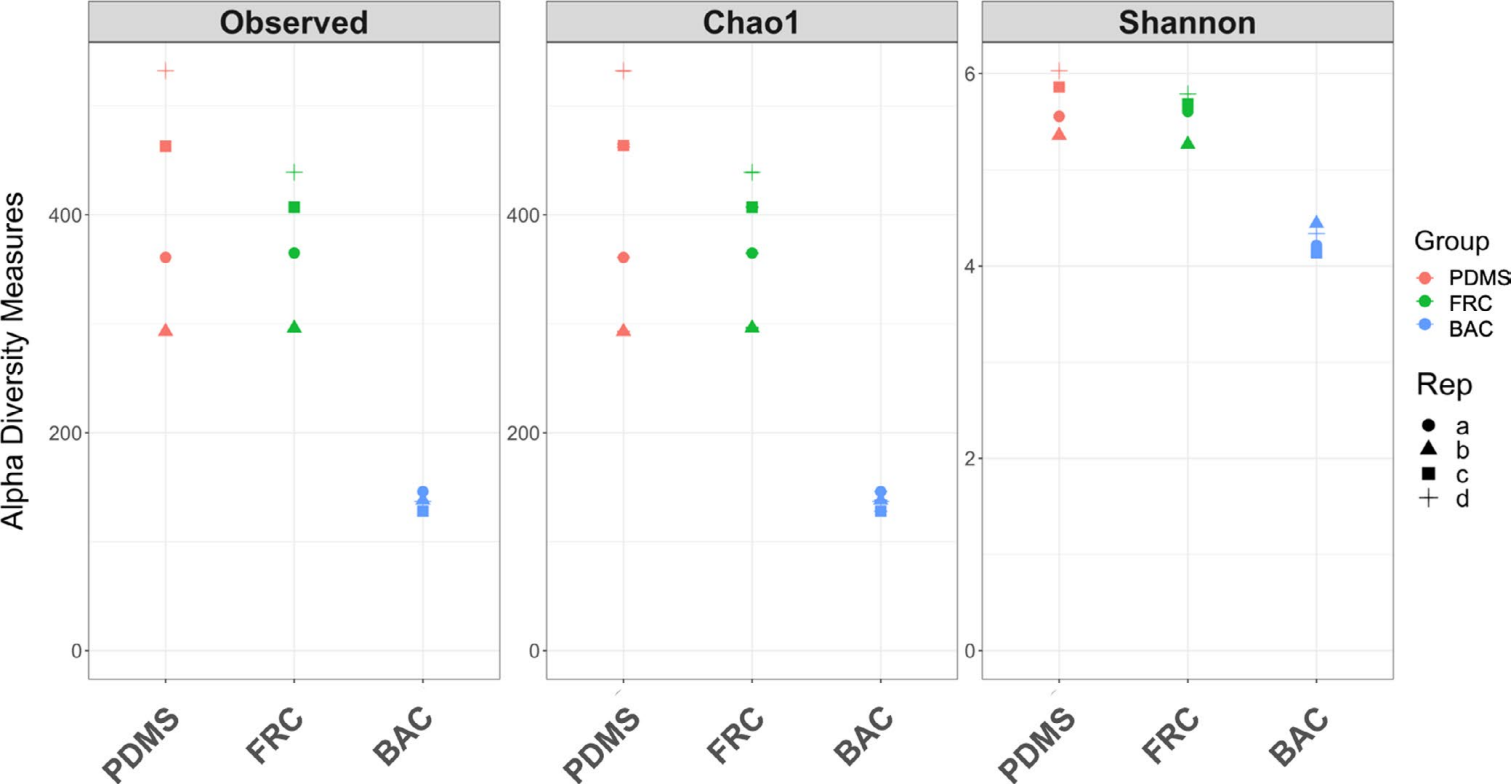
Alpha diversity estimates including the observed (unique OTUs), Chao1, and Shannon indices. Alpha diversity scores are plotted for the four replicates of each coating type. Samples are colored by coating type, each of the four replicates is indicated by a different symbol

The alpha diversity plots (for each index) of the relative abundance across OTUs for each replicate coating type (Figure 1) reflected the diversity indices estimations based on sub‐sampled OTU depths (Table 2). BAC replicate samples were the lowest, while replicates of PDMS and FRC samples had higher and closer measurements.

#### 3.2.2 Beta diversity

**FIGURE 2:**
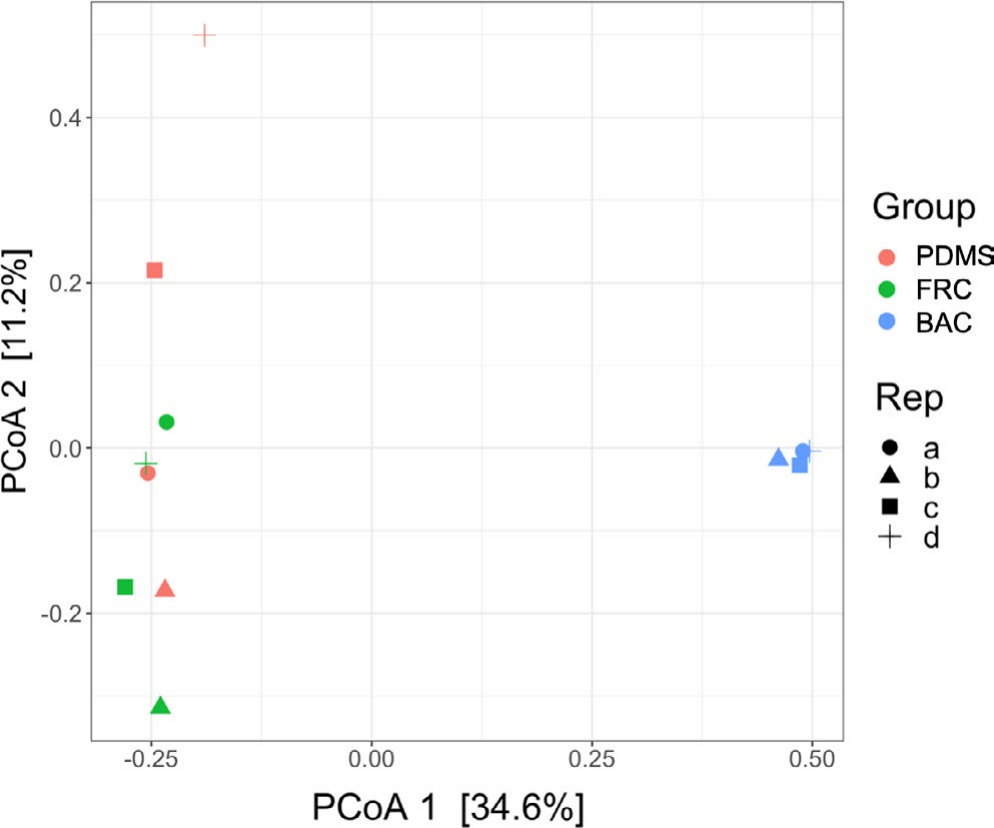
Principal coordinates analysis (PCoA) plot of the relative abundance across OTUs for each coating sample including PDMS, FRC, and BAC from 16S rRNA amplicon sequencing analysis. Variations in the dataset are explained by 34.6% with the first principal coordinate axis (PCoA 1) and 11.2% with the second axis (PCoA 2)

The principal coordinates analysis (PCoA) plot of the relative abundance of OTUs across the dataset revealed distinct communities in BAC samples, while FRC and PDMS biofilm communities showed significant overlap (Figure 2). This PCoA plot captures 45.8% of the variation in relative abundance across the dataset, with differences between BAC and both FRC and PDMS samples accounting for the majority (34.6%).

#### 3.2.3 Core biofilm microbiome

**FIGURE 3:**
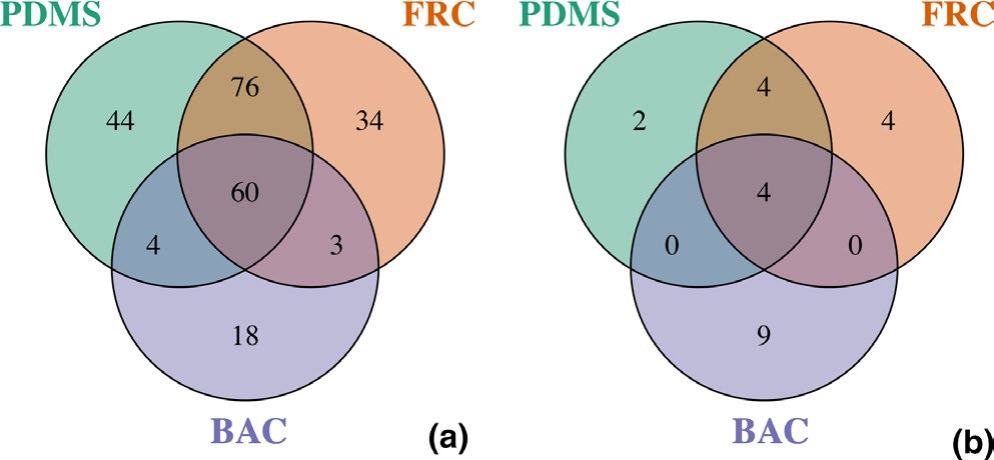
Venn diagrams representing the number of unique genera identified across OTUs identified with a relative abundance greater than (a) 0% or (b) 1% on each coating type from 16S rRNA amplicon sequencing. The overlap represents genera seen amongst the community of multiple surfaces

Particular groups that contribute to similarities (shared) and differences (distinct) between treatments were quantified and illustrated at the genus level. More specifically, OTU genera are shown with a 0% threshold regardless of their abundance in the dataset (Figure 3a), and genera with at least 1% abundance are also shown in the dataset (Figure 3b).

A total of 60 genera were shared between all samples despite their abundance as shown in Figure 3a, while a higher number of genera (76) were found shared between FRC and PDMS biofilms, suggesting that community structures of these two surfaces were similar. Mirroring the alpha diversity patterns, distinct biofilm genera on FRC (34) and PDMS (44) samples were more diverse, contrary to BAC samples which contained only 18 separate genera.

In terms of abundant genera (representing ≥1% of the community), only 4 taxa were seen in common between all treatments, while the BAC samples showed the greatest number of surface‐specific genera with 9 (Figure 3b). Therefore, the biofilm community present in BAC samples potentially contributed to the total dataset with less diverse but highly abundant genera (9 out of 18), as highlighted by the low alpha diversity measures (Table 2).

Overall, the core community of unique OTUs shared between all samples consisted of diverse genera (60) (Figure 3a), with only a small fraction of them (4) contributing with 1% abundance to the core community of abundant OTUs (Figure 3b). These results signify that the differences between surface types are defined from a few taxa that are abundant in this biofilm community.

**FIGURE A4:**
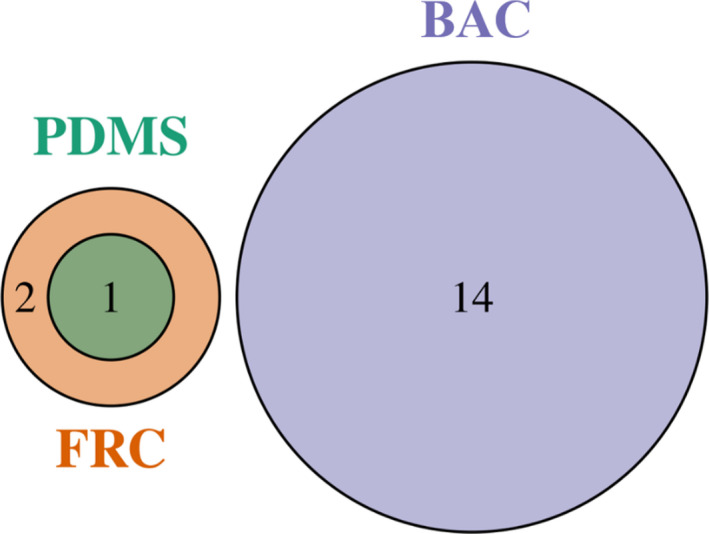
Venn diagram showing the overlap between PDMS, FRC, and BAC surfaces at the OTU level with greater than 1% relative abundance from 16S rRNA amplicon sequencing

To confirm that shared genera between surfaces were not the result of contamination between samples, a similar plot was generated for all three surfaces at the OTU level with greater than 1% relative abundance (Appendix Figure A4). At the OTU level, only a single OTU showed similar abundance across PDMS and FRC surfaces, with all other OTUs showing surface‐specific abundance, indicating that systematic contamination is unlikely.

### 3.3 Biofilm taxonomic composition explored with 16S rRNA gene marker

The biofilm taxonomic analysis revealed 24 phyla, 39 classes, 110 orders, 149 families, and 206 genera present across the three surfaces. Community composition was calculated based on percentages of the total OTUs, and below the relative abundant top taxa are presented for different taxonomic levels.

#### 3.3.1 Prokaryotic biofilm composition at the class level

**FIGURE 4:**
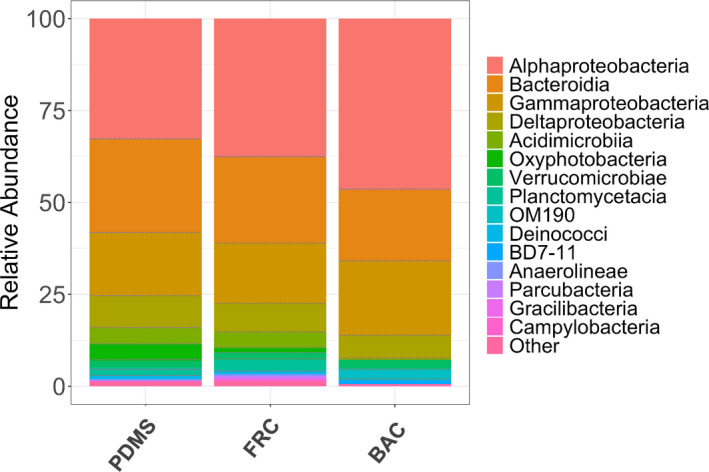
Relative abundance (%) of the top 15 abundant bacterial classes present in all biofilm samples of the PDMS, FRC, and BAC surfaces using combined replicates

Using relative abundance comparisons, the biofilms in FRC and BAC samples displayed different microbial compositions at the class level (Figure 4). Alphaproteobacteria and Bacteroidia were found consistently high across all samples, followed by Gammaproteobacteria and Deltaproteobacteria. In the biofilm community profiles of FRC and PDMS samples, Acidimicrobiia and Oxyphotobacteria (phylum Cyanobacteria) were prevailing, whereas OM190 (phylum Planctomycetes) and BD7‐11 (phylum Planctomycetes) were found enriched only in BAC samples.

#### 3.3.2 Prokaryotic biofilm composition at the genus level

**FIGURE 5:**
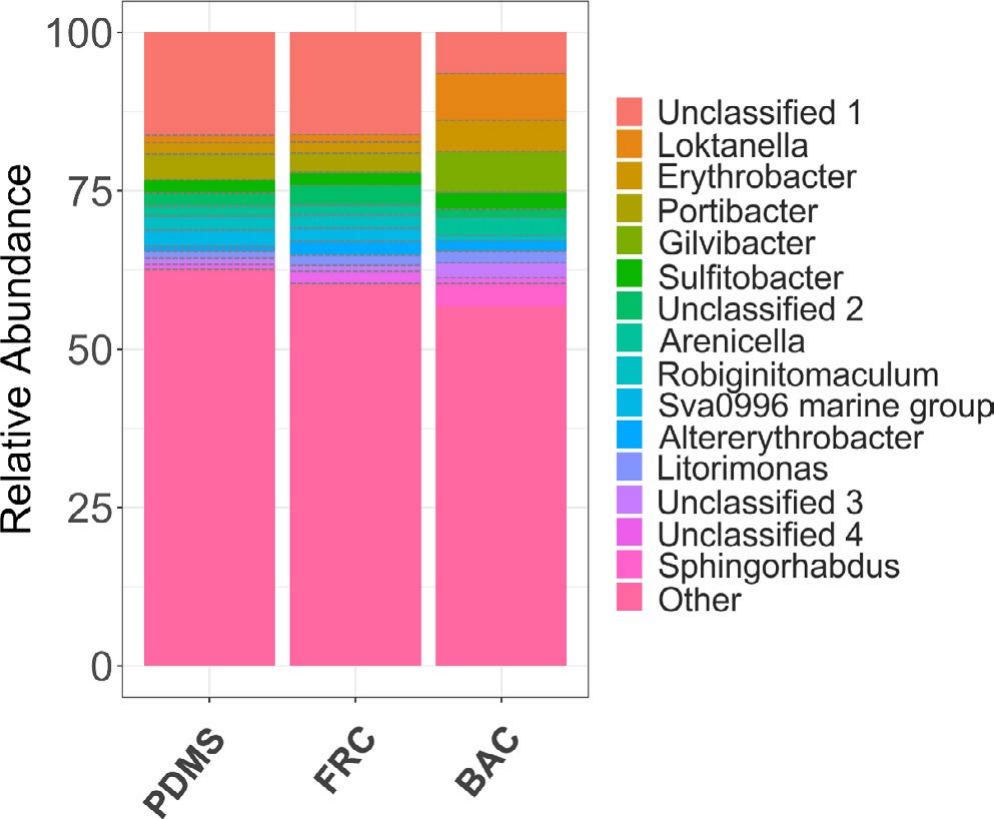
Visualization of the taxonomic profile based on the relative abundance (%) of the top 15 abundant genera in all biofilm samples isolated from PDMS, FRC, and BAC using combined replicates

The biofilm taxonomic profile of the 15 most dominant genera was further demonstrated at the genus level (Figure 5). Using relative abundance, the most prevalent genera in BAC biofilms were *Loktanella* (7.4%), *Gilvibacter* (6.4%), *Erythrobacter* (5%), *Sphingorhabdus* (3.7%), *Sulfitobacter* (2.7%), and *Arenicella* (2.6%), while other unclassified genera contributed to 6.4%. The most abundant genera in FRC samples were *Portibacter* (2.9%), Sva0996 marine group (2.2%), *Robiginitomaculum* (2.1%), and *Altererythrobacter* (2%), with 16.2% to be attributed to unclassified genera. The dominant genera in the PDMS untreated surface biofilms were *Portibacter* (4.1%), Sva0996 marine group (2.5%), *Robiginitomaculum* (2.1%), *Sulfitobacter* (2.1%), and 16.3% of unclassified genera. Overall, the FRC and PDMS samples exhibited similar biofilm community profiles compared to the BAC, although the most profound differences were the higher *Altererythrobacter*, and *Litorimonas* and smaller *Portibacter* percentages in the FRC samples.

The most pronounced differences between BAC and FRC communities were the dominance of *Loktanella* (class Alphaproteobacteria), *Erythrobacter* (class Alphaproteobacteria), *Gilvibacter* (class Bacteroidia), and *Sphingorhabdus* (class Alphaproteobacteria) in BAC, while *Portibacter* (class Bacteroidia), *Robiginitomaculum* (class Alphaproteobacteria), and Sva0996 marine group (class Acidimicrobiia) were prevailing in FRC (Figure 5). The community profile in BAC contrary to PDMS samples increased relative abundance of the genera of *Loktanella*, *Erythrobacter*, *Gilvibacter*, *Arenicella*, *Altererythrobacter*, *Litorimonas*, and *Sphingorhabdus* and decreased relative abundance of *Portibacter*, *Robiginitomaculum*, and Sva0996 marine group in BAC samples.

### 3.4 Genera significantly contributing to between‐community differences

Biofilm community structure was profiled based on the Bray‐Curtis dissimilarity metric and examined with ANOSIM to identify significant differences between coating types at the phylum (R 0.604, *p* = 0.0007***), class (R = 0.509, *p* = 0.006), family (R = 0.787, *p* = 0.002) and genus (R = 0.75, *p* = 0.000***) levels.

The genera that significantly contribute to these differences in beta‐diversity among coating types were determined by SIMPER analysis (Table 3). In total, 24 OTU biofilm genera changed with coating type (SIMPER contribution >1.5%, Kruskal *p* value <0.05). Statistical differences were significantly driven by Loktanella (*p* = 0.02), Gilvibacter (*p* = 0.02), Erythrobacter (*p* = 0.02), Portibacter (*p* = 0.02), Sva0996 marine group (*p* = 0.02), Sphingorhabdus (*p* = 0.01), and several unclassified genera.

**TABLE 3:** The significant contribution (SIMPER % >1.5%, Kruskal *p*‐value <0.05) of biofilm genera to the total similarity percentages between the different coatings revealed with SIMPER analysis

Genus	Comparisons					
	BAC/FRC		BAC/PDMS		FRC/PDMS	
	SIMPER %	*p* value	SIMPER %	*p* value	SIMPER %	*p* value
Unclassified 1	9.27	0.02	9.41	0.02	4.03	1^a^
*Loktanella*	6.22	0.02	6.15	0.02	‐	‐
*Gilvibacter*	6.00	0.02	6.12	0.02	‐	‐
*Sphingorhabdus*	3.64	0.01	3.60	0.01	‐	‐
*Erythrobacter*	3.14	0.02	3.25	0.02	1.89	0.77 ^a^
*Portibacter*	2.68	0.02	3.84	0.02	2.52	0.04
Unclassified 5	2.29	0.02	2.25	0.02	‐	‐
Sva0996 marine group	2.04	0.02	2.60	0.02	1.94	0.25 ^a^
*Aquimarina*	1.95	0.01	1.54	0.02	‐	‐
*Lentimonas*	1.80	0.08 ^a^	1.91	0.02	‐	‐
Unclassified 2	1.71	0.02	‐	‐	2.31	0.04
*Dokdonia*	1.66	0.02	2.11	0.02	1.05 ^a^	0.08 ^a^
*Peredibacter*	1.64	0.02	1.69	0.01	‐	‐
*Marinobacter*	1.55	0.01	1.53	0.01	‐	‐
*Altererythrobacter*	1.33 ^a^	0.56 ^a^	1.13 ^a^	0.25 ^a^	3.18	0.25 ^a^
*Amylibacter*	1.27 ^a^	0.39 ^a^	‐	‐	3.09	0.15 ^a^
Phormidesmis ANT.LACV5.1	‐	‐	2.08	0.02	2.91	0.02
*Litorimonas*	1.20 ^a^	0.77 ^a^	‐	‐	2.56	0.25 ^a^
Unclassified 4	1.17 ^a^	0.08 ^a^	‐	‐	2.51	0.15 ^a^
*Arenicella*	1.28 ^a^	0.15 ^a^	1.01 ^a^	0.25 ^a^	2.19	0.39 ^a^
Schizothrix LEGE 07164	‐	‐	1.38 ^a^	0.02	2.01	0.08 ^a^
OM27 clade	1.10 ^a^	0.15 ^a^	‐	‐	1.82	0.77 ^a^
*Rubidimonas*	‐	‐	1.24 ^a^	0.01	1.79	1 ^a^
*Lewinella*			1.53	0.01	1.60	0.15 ^a^

^a^ These values indicate lower (SIMPER <1.5%) or not significant (Kruskal *p* value >0.05) contribution (%).

## 4 DISCUSSION

In the present study, marine biofilms developed on two commercial fouling control coatings were examined. Biocidal antifouling and fouling‐release coated panels were sampled following a four‐month sea immersion period and analyzed using Illumina Miseq sequencing targeting the V4‐V5 region of the prokaryotic 16S rRNA gene. The age of the biofilm was previously shown to be positively associated with the number of taxa settled (Huggett et al., 2009; Winfield et al., 2018). Additionally, the “BAC” (Intersmooth® 7460HS SPC) can be specified for use with in‐service lifetimes of up to 90 months (see product description here). Therefore, the extended exposure of 119 immersion days was deliberately chosen.

### 4.1 Reported marine biofilm taxonomic profiles on fouling control coatings

**FIGURE A5:**
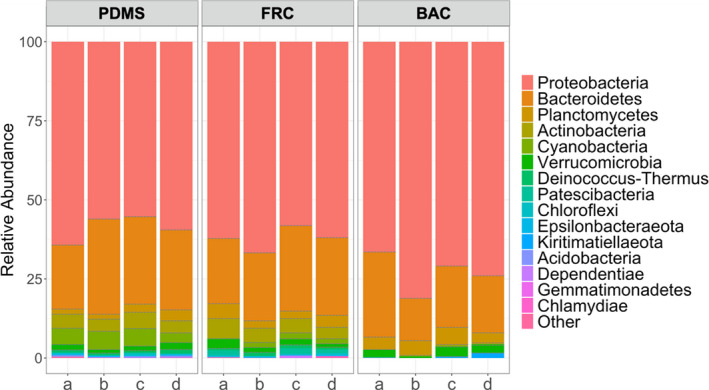
Relative abundance (%) of the top 15 abundant bacterial phyla present in all biofilm replicate samples of the PDMS, FRC, and BAC surfaces

The dominant phyla of the examined marine biofilms on the panels coated with two commercial fouling control coatings (BAC, FRC) and one inert surface (PDMS) were Proteobacteria, Bacteroidetes, Planctomycetes, Actinobacteria, Cyanobacteria, and Verrucomicrobia (Appendix Figure A5). Bacteria belonging to the classes of Alphaproteobacteria (33–47%), Bacteroidia (19–25%), and Gammaproteobacteria (16–20%), were the most dominant across all samples (Figure 4), individually contributing to more than 16% of the total biofilm community for each coating type. Deltaproteobacteria (6–9% each treatment) and Verrucomicrobiae (2% each treatment) were also dominant and present in all biofilms. When comparing with PDMS, Oxyphotobacteria, Acidimicrobiia, Planctomycetacia were similarly abundant (>1%) in FRC samples but less pronounced (<0.5 – 0.1%) in BAC samples. In all taxonomic rankings, the lowest abundance of other taxa was reported in BAC biofilms, which is potentially due to the lowest OTU diversity in BAC samples compared to the other surfaces.

The relative abundance for bacterial phyla observed in this 16S rRNA gene amplicon study is in line with the metagenomic studies of Leary et al. (2014), and Ding et al. (2019). Proteobacteria, Bacteroidetes, and Cyanobacteria have been repeatedly reported in biofilms sampled from fouling control coated surfaces (Ding et al., 2019; Hunsucker et al., 2018; Leary et al., 2014; Muthukrishnan et al., 2014). Planctomycetes (classes of BD7‐11 and OM190) that were found abundant in the biofilms sampled from all three surface types (>2.5%) in this study have also been previously recorded on fouling control surfaces (von Ammon et al., 2018; Ding et al., 2019; Leary et al., 2014), although this phylum is underestimated by previous NGS biofilm studies on fouling control coatings (e.g., Briand et al., 2017; Dobretsov et al., 2019; Flach et al., 2017; Hunsucker et al., 2018; Muthukrishnan et al., 2014; Winfield et al., 2018). Although not frequently reported, Verrucomicrobia has also been found in fouling control studies (Leary et al., 2014; Winfield et al., 2018) and was confirmed to be abundant in all three coating treatments (>1.8%) in this study.

The dominant genera (>2% of each genus) shared between the marine biofilms sampled in this study differed with coating type. The community developed on the PDMS surface was dominated by *Portibacter*, Sva0996 marine group, *Robiginitomaculum*, Phormidesmis ANT.LACV5.1, *Sulfitobacter*, and unclassified clades. Similarly, the taxonomic profile of the most abundant genera in FRC samples was characterized by *Portibacter*, Sva0996 marine group, *Robiginitomaculum*, *Altererythrobacter*, and unclassified clades. A different taxonomic profile in BAC samples reported the preeminence of *Loktanella*, *Gilvibacter*, *Erythrobacter*, *Sphingorhabdus*, *Sulfitobacter*, *Arenicella*, *Dokdonia*, *Lentimonas*, *Aquimarina*, and unclassified clades. The high abundance of other taxa at the genus level could either be attributed to the presence of diverse rare taxa or to the lack of alignment of certain taxa in the database, however, that was not observed at a higher taxonomic level (Figure 4).

The genus *Portibacter* (family Saprospiraceae), which was abundant in both PDMS and FRC samples, belongs to the phylum Bacteroidetes which is characterized by wide distribution in a variety of ecosystems, the capacity for breaking down a diverse range of organic biomacromolecules, and the preference of growing attached to surfaces (Bauer et al., 2006; Fernández‐Gómez et al., 2013). The genus *Sulfitobacter* that was found abundant in all samples in the present study regardless of coating treatment (1.9% ‐ 2.7%) has also been recorded abundant (1.05%) by Leary et al., (2014), in biofilm samples collected after 7 months from a moving ship coated with Interspeed® 640, a commercial biocidal antifouling coating that contains cuprous oxide as a biocide.

*Gilvibacter*, which was abundant in biofilms from BAC samples used in the present study (Intersmooth® 7460HS SPC which contains cuprous oxide and copper pyrithione), was previously reported by Muthukrishnan et al. (2014) as a genus found only in biofilms sampled from panels coated with biocidal antifouling Intersmooth® 360 SPC (which contains cuprous oxide and zinc pyrithione), and not biofilms sampled from Intersmooth® 7460HS SPC panels tested alongside. *Gilvibacter* was also shown to greatly contribute to dissimilarities between bacterial communities developed on other biocidal antifouling coatings attached to a coated ocean glider, such as Hempel Olympic 86950 (containing cuprous oxide and zineb) and International® Micron® Extra YBA920 (containing cuprous oxide and dichlofluanid) (Dobretsov et al., 2019). Sequences belonging to the genus *Erythrobacter*, which were found in high abundance in this study (1.8% – 5%), were previously identified on biofilms from two moving ships traveling from Norfolk North and Baltic Seas (7.7%), and Norfolk to Rota, Spain (21.3%) (Leary et al., 2014), as well as in biofilms on panels coated with cuprous oxide‐containing antifouling paints (Muthukrishnan et al., 2014) and biofilms on a coated ocean glider off the coast of Muscat, Oman (Dobretsov et al., 2019). *Sphingorhabdus* (class Alphaproteobacteria, family Sphingomonadaceae) was also abundant in BAC samples (3.6%); nevertheless, it was absent from the PDMS or FRC samples.

### 4.2 Differences between the biofilm communities on BAC and FRC coatings

The biofilm community profiles in the present study revealed major differences in OTU relative abundance and richness between the two fouling control coating treatments. Biofilm community structure was found significantly different between BAC and FRC samples for all taxonomic levels tested with ANOSIM. The differences between sample communities on the two fouling control coatings that resulted from SIMPER analysis (Table 3) were mainly driven by *Loktanella*, *Gilvibacter*, *Sphingorhabdus*, *and Erythrobacter*; sequences with high similarity to these taxa were found abundant in BAC samples, as shown in Figure 5. Additionally, SIMPER analysis illustrated that *Portibacter* and Sva0996 marine groups which were abundant on the FRC surface (Figure 5), constituted key components defining the different community profiles between the two fouling control coatings.

Biofilm communities found on FRC panels were similar to those on PDMS surfaces, while BAC biofilms exhibited a distinct response, as indicated by sample clustering in the PCoA plot (Figure 2). The highest biofilm diversity indicated by all diversity indices (Table 2) was found on the PDMS and FRC surfaces. The biofilm profile of BAC panels was characterized by a lower diversity and a higher relative abundance of the present taxa.

In terms of the BAC biofilm community profile, the higher relative abundance may be due to the relative proliferation of a few biocide‐tolerant taxa or may be a result of species competition which shifted the community composition. The observed relatively high abundance of few taxa in BAC biofilms is consistent with earlier (microscopic) investigations of biofilm composition and relative abundance in samples from fouling release and biocidal antifouling‐coated surfaces that revealed lower abundance and higher diversity in samples from fouling‐release surfaces (Cassé & Swain, 2006). The lower diversity observed here in BAC samples could be attributed to the effect of biocides in inhibiting the settlement of certain taxa that exhibit sensitivity toward biocidal toxicity, such as *Portibacter* (0.09%) or Sva0996 marine group (0.06%) that were almost absent in BAC samples. Conversely, the highest relative abundance reflected by the contribution of dominant taxa to the overall community of each sample was detected in BAC samples, followed by FRC and PDMS. Potential biocidal tolerance could be reflected by changes in relative abundance evident for the class of BD7‐11 (phylum Planctomycetes) that was absent in biofilms sampled from the other two coatings (BAC: 1.0%, FRC, PDMS: 0%). Additionally, the genera found present on BAC samples and missing on FRC were as follows: *Sphingorhabdus* (BAC: 3.7%, FRC: 0%), *Aquimarina* (BAC: 2%, FRC: 0%), *Marinobacter* (BAC: 1.6%, FRC: 0%), HTCC5015 (BAC: 1.6%, FRC: 0%), and *Maribacter* (BAC: 0.7%, FRC: 0%).

Alphaproteobacteria that dominated BAC surface biofilms (i.e., *Loktanella*, *Erythrobacter*, *Sphingorhabdus*) were different from those dominating FRC surface biofilms (i.e., *Robiginitomaculum*), which is a possible indication of diverse synergistic relationships between abundant bacteria present in these biofilms. Cyanobacteria have been suggested to exhibit high resistance to heavy metals leaching out of biocidal antifouling coatings (Cassier‐Chauvat & Chauvat, 2015) and were previously reported abundant on biocidal antifouling‐coated surfaces (Leary et al., 2014; Muthukrishnan et al., 2014). The present study shows the opposite since Cyanobacteria (class Oxyphotobacteria) detected sequences dominated PDMS (4.6%) and FRC (1.39%) surfaces, contrary to BAC (0.1%). It has to be noted that high dominance of Cyanobacteria on BAC coatings has been suggested after 1 year of immersion in Oman (Muthukrishnan et al., 2014) and after 7 months on two moving vessels crossing the North and Baltic Seas, and North‐East Atlantic Ocean, respectively (Leary et al., 2014). Here, Cyanobacteria were not abundant on BAC that was exposed for 4 months in Langstone Harbour, UK.

Certain bacterial genera such as *Loktanella* and *Gilvibacter*, which possibly exhibit tolerance to biocides contained in BAC, potentially reduced the settlement or growth of other organisms on BAC that were abundant in the other two surfaces (e.g., *Portibacter*) (Figure 5). In comparison with the PDMS, *Portibacter* was the only bacterial genus where the relative abundance was reduced in both coatings, BAC and FRC. On the BAC panels, two factors that are possibly involved in shaping the shifted community are the performance of the biocidal paint and the interplay between biofilm components at certain conditions (e.g., biocidal release rate, environmental conditions, antagonistic relationships).

### 4.3 Study design suggestions for biofilm research on fouling control surfaces

The current study has carefully implemented the most relevant design (four biological replicates were tested, with immediate biofilm storage in liquid nitrogen, targeting the V4‐V5 region of 16S rRNA gene, using Illumina MiSeq NGS technology, sequence annotation against the SILVA SSU 132 database, etc.) to support the purpose of the study, as many factors during experimental design and data analysis could significantly impact the results—especially in a complex microbial community.

The V4‐V5 region of the 16S rRNA gene has been one of the most broadly used variable regions in studies examining environmental biofilms on artificial surfaces (e.g., Bakal et al., 2018; Li et al., 2017; Pereira et al., 2017), while 515F/926R has been suggested as a primer set that increases percentage detection of various prokaryotic taxa (Pollet et al., 2018) as well as been the most effective region in minimizing overestimation due to intragenomic heterogeneity (Sun et al., 2013).

For microbial community analyses, Illumina is the most widely used NGS platform, due to the large output and cost performance (van Dijk et al., 2018; Fukuda et al., 2016) which are indispensable in complex and diverse study systems. Illumina produces high throughput and short read length with a low error rate (de Sá et al., 2018).

The selection of 16S rRNA sequence reference database is an important element for taxonomic classifications; therefore, it is worth mentioning that only Briand et al., (2017) have used the SILVA SSU database similar to the present study, while other studies of biofilms on fouling control have used the RDP (von Ammon et al., 2018; Dobretsov et al., 2019; Muthukrishnan et al., 2014) or Greengenes (Hunsucker et al., 2018; Winfield et al., 2018) databases. The SILVA database constitutes one of the most actively maintained and largest databases which includes curated 16S rRNA gene sequences (Quast et al., 2013; Yilmaz et al., 2014), while it has been suggested that it provides the lowest error rates compared to Greengenes and RDP (Lu & Salzberg, 2020).

It is worth highlighting that in biofilm studies on fouling control it is difficult to examine a “true” control to enable understanding the effect of specific coatings to the already‐existing communities due to the extent of macrofouling. Moreover, the free‐living microorganisms in the surrounding seawater at the time of sample collection could not serve as an indicator sample for comparison with mature biofilms developed on fouling control surfaces. The limited number of studies employed to date has examined biofilm composition on different types of fouling control coatings without testing a reference surface (Hunsucker et al., 2018; Winfield et al., 2018). It is also worth noting that a lack of negative controls in the present study represents a limitation of the study design since the presence of systematic contamination from the extraction and PCR stages cannot be identified and accounted for. However, the lack of systematic OTU abundance across the surfaces (Appendix Figure A4) suggests that contamination was not present at levels likely to affect the differential abundance analysis between the three major surfaces considered in this study.

In the present study, the generic unmodified PDMS coating was included as an inert surface to reflect the representative biofilm communities under the given conditions (e.g., location, season). Unmodified PDMS is not suitable for commercial use as a fouling control product. However, it shares some surface characteristics with fouling‐release coatings as an elastomeric material with a very smooth surface profile, and it demonstrates greater resistance to macrofouling compared to other unprotected artificial surfaces which is a useful pragmatic property for field studies. It is therefore advantageous to incorporate a non‐toxic, inert, and macrofouling‐resistant surface in fouling control research studies to (1) improve understanding of the microfouling communities that form with respect to coating properties, (2) better contextualize similarities and differences that arise between the complex biofilm communities that develop on different surfaces, and (3) discover the potential interplay between biofilm taxonomic components.

It is important to highlight that the fouling control coatings used in this study are designed primarily for use on the world's commercial shipping fleet, whose operational profiles typically involve alternating static periods in and around port and periods of active movement at sea. As expressed by Davidson et al., (2020), lay‐up periods are common, inevitable, unavoidable, and of high significance because of the potential for fouling establishment. In their study of simulated lay‐up periods followed by resumption of service, panels coated in biocidal and fouling release coatings showed similar levels of low fouling after exposure to flow at the lower range of ship speeds. The final biofouling loads were attributable to lower initial fouling on the biocidal surface and the loss of fouling that had accrued on the fouling release surfaces, consistent with the modes of actions of the coating types (Davidson et al., 2020). On the other hand, in high flow conditions, different microbial community profiles would not be surprising. As hydrodynamic conditions interact with fouling control coatings through biocidal release and/or stressing fouling adhesion, and modify community profiles (Cassé & Swain, 2006; Krsmanovic et al., 2021), it would be expected that the presence of flow might accentuate biofilm community divergences driven by underlying coating properties. Alternatively, the additional environmental stress of high shear might result in the convergence of biofilm communities toward a common pool of organisms with strong adhesion properties. Dynamic immersion or ship‐based studies in the future might address these questions. Given the substantially static conditions under which the test panels were deployed in the present study (tidal‐flow only), the current research outcomes are indicative of the compositional and relative abundance differences of marine biofilms that develop during idle periods on coated toxic and non‐toxic surfaces with divergent material properties and could be used as a guide in future experiments.

## 5 CONCLUSION

The present investigation has added to the growing body of biofilm studies on fouling control coatings using NGS analysis, demonstrating that fouling control coating properties can significantly influence microfouling development. Distinct biofilm profiles were reported between the three coating types: the biocidal antifouling coating “BAC” displayed higher abundance and lower diversity compared to the other two surfaces, while in contrast, the fouling‐release coating “FRC” showed strong similarities with the generic unmodified “PDMS” coating. The biocides contained in the examined BAC coating (Intersmooth® 7460HS SPC) were cuprous oxide and copper pyrithione and demonstrated a clear impact on the biofilm community composition.

Even though biocidal antifouling coatings largely prevent macrofouling, they also lead to the development of very different biofilm communities. The biofilm community that develops on biocidal coating surfaces may encompass important components with specialized behavior driven by their unique genes. The outcomes of the current study suggest that Alphaproteobacteria (genus Loktanella, Sphingorhabdus, and Erythrobacter) and Bacteroidetes (genus Gilvibacter) may exhibit high tolerance to the biocide flux emanating from BAC Intersmooth® 7460HS SPC under the test conditions that were deployed. Potential lack of biocidal tolerance and selective attachment on FRC Intersleek® 900 is suggested for a group of Bacteroidetes (genus Portibacter) and Actinobacteria (genus Sva0996 marine group). Reporting key biofilm components with tolerance to biocides and exploring the gene expression of these versatile communities is fundamental for controlling microfouling.

To realistically eradicate toxic biocides from fouling control paints, effective and robust alternatives must be developed. In this study, it was shown that the examined FRC did not have a large effect on biofilm composition and relative abundance when compared to an inert surface (i.e., PDMS). However, fouling‐release coatings should also be tested under dynamic conditions which more closely reflect their expected in‐service exposure conditions, while the largely static conditions in the current study were not representative of a moving vessel (tidal movement only). Future investigations may shed light on the gene expression profiles of these complex biofilm communities and identify key genes that contribute to efficiency against biocides. The examination of biofilms formed on commercial fouling control coatings used in ship's hulls will provide keystone information to scientists and manufacturers in designing more robust and environmentally compatible fouling control systems. The outcomes of this project are anticipated to have important implications for the future development of novel fouling control surfaces.

## CONFLICTS OF INTEREST

None declared.

## AUTHOR CONTRIBUTIONS

**Maria Papadatou:** Data curation (equal); Formal analysis (supporting); Investigation (lead); Methodology (equal); Visualization (lead); Writing‐original draft (lead); Writing‐review & editing (equal). **Samuel C. Robson:** Data curation (equal); Formal analysis (lead); Software (lead); Supervision (supporting); Writing‐review & editing (equal). **Sergey Dobretsov:** Funding acquisition (supporting); Writing‐original draft (supporting); Writing‐review & editing (supporting). **Joy E.M. Watts:** Writing‐review & editing (supporting). **Jennifer Longyear:** Resources (supporting); Writing‐review & editing (supporting). **Maria Salta:** Conceptualization (lead); Funding acquisition (lead); Investigation (supporting); Methodology (equal); Project administration (lead); Supervision (lead); Writing‐review & editing (equal).

## ETHICS STATEMENT

None required.

## Data Availability

The dataset generated and/or analyzed during this study are available in the NCBI Sequence Read Archive (SRA) repository under BioProject accession number PRJNA728737: https://www.ncbi.nlm.nih.gov/bioproject/PRJNA728737.

## APPENDIX 1

**TABLE A1:** Characteristics of the commercial fouling control coatings examined. BAC: commercial biocidal antifouling; FRC: fouling‐release coating; PDMS: polydimethylsiloxane

Sample type	Surface technicalities	Active Ingredients (Biocides)
BAC	Self‐polishing copolymer (SPC) *Intersmooth® 7460HS SPC*	Cuprous oxide +CuPT (copper pyrithione)
FRC	Fluoropolymer finish *Intersleek® 900*	None
PDMS	Silicon‐based organic polymer	None
